# Increased serum 12-hydroxyeicosatetraenoic acid levels are correlated with an increased risk of diabetic retinopathy in both children and adults with diabetes

**DOI:** 10.1007/s00592-022-01951-7

**Published:** 2022-08-12

**Authors:** Shuli Chen, Yu Qian, Qiurong Lin, Zhangling Chen, Zhaoyu Xiang, Lipu Cui, Jiaqi Sun, Xinran Qin, Yi Xu, Lina Lu, Haidong Zou

**Affiliations:** 1 Department of Ophthalmology, Shanghai General Hospital, Shanghai Jiao Tong University, School of Medicine, No.100 Haining Road, Shanghai, 200080 China; 2 Shanghai Eye Disease Prevention and Treatment Center/Shanghai Eye Hospital, Shanghai, China; 3 Shanghai Engineering Center for Precise Diagnosis and Treatment of Eye Diseases, Shanghai, China; 4 Department of Ophthalmology, Shanghai General Hospital of Nanjing Medical University, Shanghai, China; 5 Department of Ophthalmology, Shanghai Songjiang District Central Hospital, Shanghai, China; 6 National Clinical Research Center for Eye Diseases, Shanghai, China

**Keywords:** Diabetes mellitus, Diabetic retinopathy, 12-HETE, Children, Adult

## Abstract

**Purpose:**

To investigate the relationship between serum 12-Hydroxyeicosatetraenoic acid (12-HETE) and diabetic retinopathy (DR) in children with type 1 diabetes mellitus (T1DM) and adults with type 2 diabetes mellitus (T2DM).

**Methods:**

Children from the Shanghai Children and Adolescent Diabetes Eye (SCADE) study and adults from the Shanghai Cohort Study of Diabetic Eye Disease (SCODE) were examined in 2021. Serum 12-HETE levels were detected and compared. Multivariate logistic regression was used to analyze the relationship between 12-HETE and the rate of DR in diabetic patients.

**Results:**

The child study included 4 patients with new-onset DR and 24 patients with T1DM without DR. In children with T1DM, the 12-HETE level was significantly higher in those with DR (*P* = 0.003). The adult study had two sets, for testing and verification. The test set included 28 patients with new-onset DR and 24 T2DM patients with a course of ≥ 20 years who had never developed DR. The verification set included 41 patients with DR, 50 patients without DR and 50 healthy controls. In the adult test set, the 12-HETE level was significantly higher in patients with DR than in those with T2DM without DR (*P* = 0.003). In the verification set, the 12-HETE level of patients with DR was significantly higher than that of patients without DR (*P* < 0.0001) and the healthy controls (*P* < 0.0001). Multivariate logistic regression indicated that 12-HETE was independently associated with DR in both children (odds ratio [OR] 1.06, 95% confidence interval [CI] 1.00–1.13, *P* = 0.041) and adults (test set [OR 9.26, 95% CI 1.77–48.59, *P* = 0.008], verification set [OR 10.49, 95% CI 3.23–34.05, *P* < 0.001]).

**Conclusion:**

Higher serum 12-HETE levels are positively correlated with an increased risk of DR in children with T1DM and adults with T2DM.

**Supplementary Information:**

The online version contains supplementary material available at 10.1007/s00592-022-01951-7.

## Introduction

As the global prevalence of diabetes continues to rise, diabetic retinopathy (DR), the most common and serious ocular complication of diabetes, has become one of the main causes of vision loss worldwide [1]. In the early stage of DR, namely, nonproliferative DR (NPDR), retinal pathologies, including microaneurysms, hemorrhages and hard exudates, can be observed. When it progresses to advanced proliferative DR (PDR), characterized by neovascularization, patients may suffer severe visual impairment [2]. The progression of DR is insidious and accompanied by irreversible retinal pathological changes, making it critical to control its risk factors and strengthen its early prevention. The currently known risk factors include hyperglycemia, hypertension, hyperlipidemia, diabetes duration, race, and genetics [3, 4]. The large individual differences in the development and severity of DR cannot be fully explained by these traditional factors, such as blood glucose, blood pressure and diabetes duration, so more biomarkers need to be identified [5].

Through the application of metabolomics, various lipid metabolism abnormalities have been found related to DR, including arachidonic acid (AA), a polyunsaturated fatty acid (PUFA) [6–10]. 12-Hydroxyeicosatetraenoic acid (12-HETE) is the main metabolite of AA, formed by reactions catalyzed by 12-lipoxygenase (12-LOX) and 12/15-LOX (15-LOX-1 in humans) [11]. Significant increases in retinal 12-HETE content have been detected in both oxygen-induced retinopathy and diabetic mouse models [12, 13]. Baicalein, a nonspecific LOX inhibitor, could prevent 12-HETE production and retinal neovascularization [12, 13]. However, levels of AA metabolites in the peripheral blood of diabetic mice were not significantly different from those of normal mice [14]. So far, there are few population studies on 12-HETE in DR. Ye et al. found that AA metabolites in the feces of PDR patients were significantly higher than those of diabetic patients without DR [15]. Lin et al. found that the levels of LOX pathway metabolites of AA in the vitreous humor of PDR patients were significantly higher than those of normal subjects, including 12-HETE [16]. A multiplatform metabolomics clinical study conducted by our group found that the serum 12-HETE level in 350 DR subjects was significantly higher than that of 111 diabetic patients without DR and predicted it to become a biomarker for DR diagnosis [17]. This has been the only study to find a correlation between serum 12-HETE and the prevalence of DR. But the clinical characteristics differed greatly between the groups, and an influence of factors such as blood glucose, blood pressure, and diabetes duration on the results could not be ruled out. No study has elucidated the relationship between 12-HETE and DR in populations with type 1 diabetes mellitus (T1DM).

To further clarify the relationship between 12-HETE and DR, this paper investigated two long-term annual follow-up eye disease cohorts previously established by this research team: children with T1DM and adults with type 2 diabetes mellitus (T2DM). The recent follow-up results in 2021 were analyzed to explore the relationship between serum 12-HETE and DR in these two populations.

## Methods

### Subjects

The children with T1DM were from the Shanghai Children and Adolescent Diabetes Eye (SCADE) study (clinicaltrials.gov identifier: NCT03666052). Included in this part of the study were a subset of children with T1DM who participated in the SCADE project in January 2019 and were followed up again in January 2021. The inclusion criteria, exclusion criteria, and examination methods of SCADE were as described [18, 19]. In short, the inclusion criteria in 2019 were as follows: (1) diagnosis of T1DM; (2) age under 18 years; (3) full cooperation with the examination; (4) best-corrected visual acuity of both eyes ≥ 0.8; and (5) clear refractive medium. The exclusion criteria were (1) presence of typical DR changes, such as retinal microaneurysm and hemorrhage; (2) history of other eye diseases, such as glaucoma, macular degeneration, or choroidal disease; and (3) history of ophthalmic surgery.

The adults with T2DM were from the Shanghai Cohort Study of Diabetic Eye Disease (SCODE) (clinicaltrials.gov identifier: NCT03665090). Since 2003, we have kept health records of diabetic residents in the community of Changning District, Shanghai, and conducted annual examinations, covering demographic characteristics, biochemical indices, and ophthalmic data [20]. The inclusion criteria, exclusion criteria, and examination methods of the SCODE study have been described elsewhere [19].

The study adhered to the ethical principles of the Declaration of Helsinki and was approved by the Ethics Committee of Shanghai General Hospital (approval number: 2013KY023, 2018KY209) and the Ethics Committee of Children’s Hospital of Fudan University (approval number: 01[2018]). Informed consent was signed by each adult subject and the legal guardian of each child.

The diagnosis of T1DM and T2DM was based on the diagnostic criteria for diabetes proposed by the WHO in 1999. DR diagnosis was based on the International Clinical Classification System for DR proposed at the International Ophthalmology Conference in 2002 [21]. Eyes with microaneurysms only were considered as mild NPDR. Severe NPDR was diagnosed when any of the following occurs without signs of PDR: (1) more than 20 intraretinal hemorrhages in each of 4 quadrants; (2) definite venous beading in 2 + quadrants; and (3)prominent intraretinal microvascular abnormalities in 1 + quadrant. Those between mild and severe NPDR were diagnosed as moderate NPDR. PDR was diagnosed when definite neovascularization was discovered.

### Clinical data

The personal information was recorded, such as date of birth, sex, height, weight, past medical history, diabetes type, and diagnosis time. Body mass index (BMI) was calculated as weight in kilograms divided by height in meters squared (kg/m^2^). Venous blood samples were drawn, and the laboratory parameters tested included fasting plasma glucose (FPG), glycated hemoglobin (HbA1c), blood lipids, liver function and renal function. All subjects underwent routine ophthalmic examinations: (1) The best-corrected visual acuity was detected using the international standard LogMAR visual acuity chart (Wenzhou Xingkang, Zhejiang, China). (2) A slit-lamp biomicroscope (SL130, Zeiss, Germany) was used to examine the eyelid, conjunctiva, cornea, anterior chamber, iris, pupil, and lens. (3) The intraocular pressure was measured with a non-contact tonometer (NT510; NIDEK, Tokyo, Japan). (4) The eye axis, corneal thickness, corneal diameter, corneal curvature, anterior chamber depth, and lens thickness were examined using an optical biometer (IOL Master 700, Zeiss, Germany). Digital fundus photography (ss-OCT; Topcon, Tokyo, Japan) under mydriasis was performed, and macula-centered and optic disc-centered colored photographs were taken for each eye. All eye examinations were performed by experienced ophthalmologists. The levels of DR were assessed by Dr. Zou and Dr. Xu.

### Measurement of 12-HETE

All serum samples were collected after overnight fasting and stored at  − 80 °C for further testing. A 90-μL aliquot of each sample was transferred to an Eppendorf tube. After the addition of 400 μL of extract solution (methanol:acetonitrile = 1:1, precooled at − 40 °C, containing isotopically labeled internal standard mixture), the samples were vortexed for 30 s and sonicated for 15 min in an ice-water bath, followed by incubating at − 40 °C for 1 h. After centrifugation (15 min, 12,000 rpm, and 4 °C), a 400-μL aliquot of the supernatant was transferred to an Eppendorf tube. Then the supernatant was evaporated to dryness under a gentle stream of nitrogen and reconstituted in 50 μL water containing 10% acetonitrile. After centrifugation (15 min, 12,000 rpm, and 4 °C), the clear supernatant was subjected to ultrahigh-performance liquid chromatography-tandem mass spectrometry (UHPLC-MS/MS) analysis. UHPLC separation was carried out using an EXIONLC System (Sciex) equipped with a Waters ACQUITY UPLC HSS T3 column (100 × 2.1 mm, 1.8 μm, Waters). Mobile phase A was water containing 0.1% formic acid and 2 mM ammonium acetate. Mobile phase B was acetonitrile containing 0.1% formic acid and 2 mM ammonium acetate. The column temperature was 40 °C. The autosampler temperature was 4 °C. The injection volume was 10 μL. A SCIEX 6500 QTRAP + triple-quadrupole mass spectrometer (Sciex) equipped with an IonDrive Turbo V electrospray ionization (ESI) interface was applied for mass spectrometry in multiple reaction monitoring (MRM) mode. Typical ion source parameters were as follows: curtain gas = 40 psi, ion spray voltage =  ± 4500 V, temperature = 500 °C, gas 1 = 30 psi, and gas 2 = 30 psi. SCIEX Analyst Work Station Software (Version 1.6.3) and Sciex MultiQuant software (Version 3.0.3) were employed for MRM data acquisition and 12-HETE quantitative analysis. Calibration solutions were subjected to UHPLC-MRM-MS/MS analysis using the methods described above. The calibration curve was drawn, where y is the ratio of the peak area of 12-HETE to that of the internal standard, and x is the ratio of the concentration of 12-HETE to that of the internal standard. The correlation coefficient of regression fit was 0.99993. The QC sample was injected with 7 technical replicates. The analytical recovery was 105.20%, and the relative standard deviation was 9.23%.The 12-HETE concentration in the samples was calculated from the calibration curve.

### Statistical analysis

Statistical analysis was performed with SPSS 26.0 and GraphPad Prism 9 software. Continuous variables are presented as mean ± standard deviation. Categorical variables are presented as frequencies (percentages). For continuous data, the Kolmogorov–Smirnov test was used to judge whether the data conformed to a normal distribution. Student’s t-test or the Mann–Whitney U test was used to compare differences between two independent groups. ANOVA with the least significant difference (LSD) post hoc tests or the Kruskal–Wallis H test was used to compare differences between three independent groups. Chi-square test was used to compare the proportions of categorical variables. A binary logistic regression model was used to analyze factors associated with DR. The variables with *P* < 0.10 in the univariate analysis were entered into the multivariate model, where the forward stepwise regression method was used. Statistical significance was assumed at *P* < 0.05 (two-tailed).

## Results

### Children study

A total of 37 children with T1DM in the SCADE cohort met the inclusion criteria in 2019, of whom 28 were followed up in 2021. The other 9 did not come for follow-up, due to the COVID-19 epidemic. In 2021, 4 of these 28 children developed DR, manifested as fundus hemorrhage or microvascular abnormalities in the retina. These 4 patients were included in the Children-DR group, and the other 24 patients, who did not develop DR, were included in the Children-non-DR (Children-NDR) group. There were no significant differences in the demographic or clinical characteristics between the two groups (Table 1).

**Table 1:** Clinical characteristics for the Children-NDR and Children-DR groups

Characteristics	Children-NDR (*n* = 24)	Children-DR (*n* = 4)	*P*-value
Age (years)	13.13 ± 2.98	11.25 ± 4.27	0.369^a^
Male, N (%)	8 (33)	3 (75)	0.269^c^
BMI (kg/m^2^)	20.81 ± 2.96	18.61 ± 2.42	0.529^a^
T1DM duration (years)	4.13 ± 2.83	4.25 ± 2.75	0.976^a^
HbA1c (%)	7.86 ± 1.50	7.63 ± 1.26	0.728^b^
SBP (mmHg)	114.21 ± 13.12	113.50 ± 14.71	0.605^a^
DBP (mmHg)	66.29 ± 9.12	74.50 ± 16.09	0.267^a^
TC (mmol/L)	4.21 ± 0.75	4.65 ± 1.33	0.340^a^
TG (mmol/L)	0.96 ± 0.79	1.08 ± 0.47	0.764^a^
HDL-C (mmol/L)	1.89 ± 0.49	1.88 ± 0.82	0.969^a^
LDL-C (mmol/L)	2.33 ± 0.64	2.75 ± 1.09	0.289^a^
12-HETE (pg/mL)	21.67 ± 16.00	83.92 ± 69.59	0.003^b^

a, Student’s t-test. b, Mann–Whitney U test. c, Chi-square test

BMI, body mass index; HbA1c, glycated hemoglobin; SBP, systolic blood pressure; DBP, diastolic blood pressure; TC, total cholesterol; TG, triglyceride; HDL-C, high-density lipoprotein cholesterol; LDL-C, low-density lipoprotein cholesterol

**Fig. 1:**
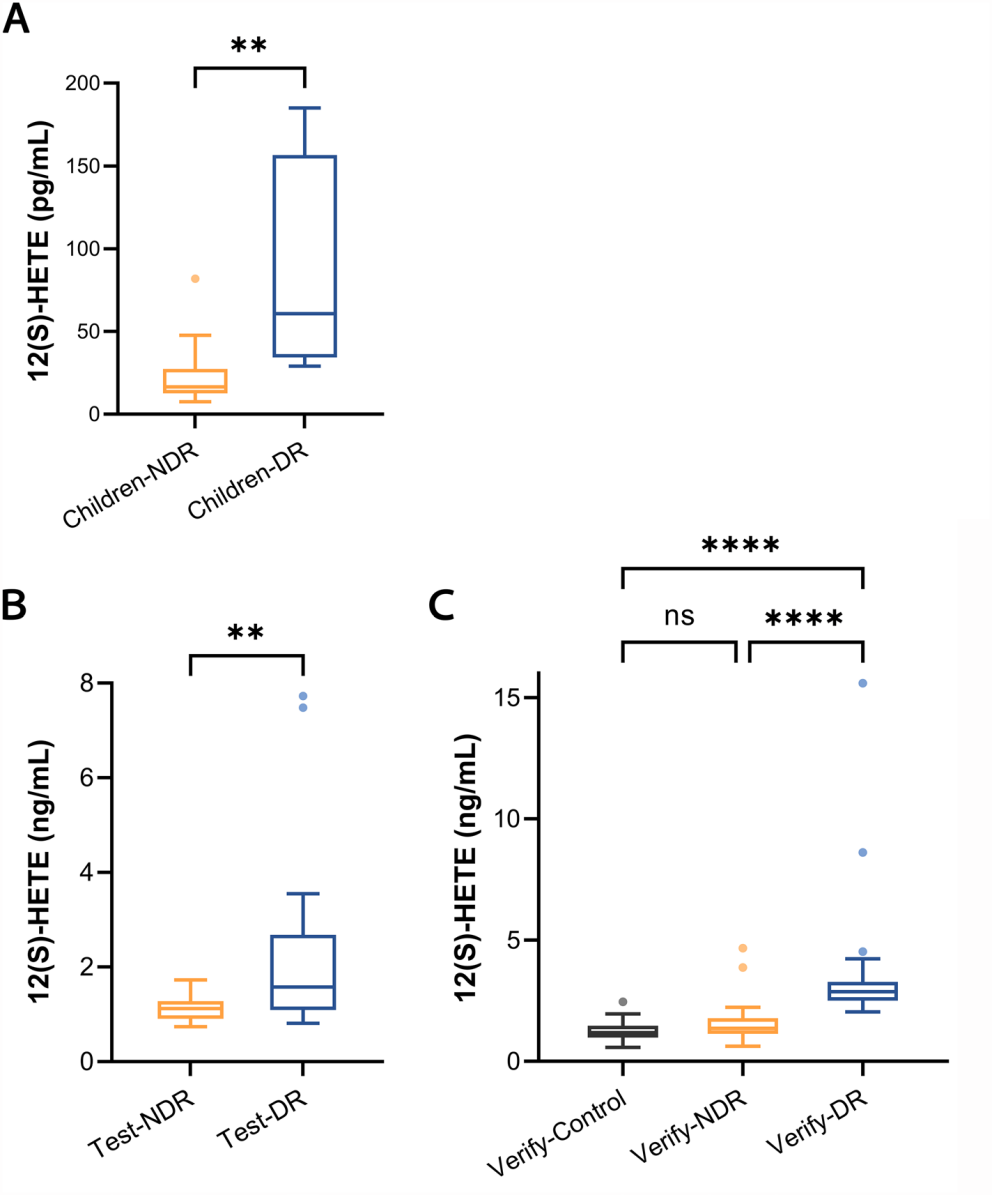
Distribution and comparison of the serum 12-HETE levels.** A** Group Children-DR and Children-NDR.** B** Group Test-NDR and Test-DR.** C** Group Verify-Control, Verify-NDR, and Verify-DR. *P*-value was estimated via Mann–Whitney U test. **P < 0.01, ****P < 0.0001

The 12-HETE level in the Children-DR group was significantly higher than that in the Children-NDR group (*P* < 0.01) (Fig. 1A). Binary logistic regression analysis was performed, where the incidence of DR was the dependent variable, and age, sex (0 = female, 1 = male), BMI, diabetes duration, HbA1c, systolic blood pressure (SBP), diastolic blood pressure (DBP), blood lipids, and 12-HETE were input as independent variables. The univariate analysis is shown in Table S1. In the end, only 12-HETE was associated with the incidence of DR, with an odds ratio (OR) of 1.06 (95% confidence interval [CI] 1.00–1.13, *P* = 0.041).

### Adult study

The study included 193 adult subjects with T2DM from the SCODE cohort. It was conducted in two steps: testing and verification.

Fifty-two adults were included in the test set, all from Xinjing Community, Shanghai. The Test-DR group included patients newly diagnosed with DR in 2021 (*n* = 28), while the Test-NDR group included those who had not developed DR despite a diabetes duration of ≥ 20 years (*n* = 24). The 141 subjects in the verification set were from Jiangsu Road Subdistrict and Xinjing Community and were divided into three groups. Of the 141 subjects, 41 patients who were previously diagnosed with DR were included in the Verify-DR group, 50 patients with a diabetes duration < 20 years who had not developed DR in the Verify-NDR group, and 50 healthy controls in the Verify-Control group.

The demographic and clinical characteristics of the subjects are shown in Table 2. The two groups of the test set were well matched, though there was a significant difference in diabetes duration owing to the specific inclusion criteria. There were some differences in the characteristics of the verification set. For some characteristics, such as HbA1c and FPG, the differences may be attributed to diabetes itself. However, the differences in some indicators, such as total bilirubin and urea, may be due to the small sample size and the potential existence of confounding factors. After adjustment in the multivariate analysis (mentioned below), the differences in these indicators were no longer significant.

**Table 2:** Clinical characteristics for the subjects in the test set and the verification set

Characteristics	Test Set			Verification Set			
	Test-NDR (*n* = 24)	Test-DR (*n* = 28)	*P*-value	Verify-Control (*n* = 50)	Verify-NDR *(n* = 50)	Verify-DR (*n* = 41)	*P*-value
Age (years)	70.13 ± 8.46	71.64 ± 7.61	0.496^a^	70.50 ± 5.78	68.62 ± 5.10	69.07 ± 9.92	0.281^e^
Male, N (%)	14 (58)	13 (46)	0.392^c^	17 (34)	31 (62)^*^	23 (56)	0.014^c^
BMI (kg/m^2^)	23.56 ± 2.91	24.77 ± 2.41	0.254^a^	24.74 ± 3.62	25.26 ± 3.74	24.20 ± 2.51	0.469^e^
T2DM duration (years)	23.39 ± 0.63	16.30 ± 1.23	< 0.001^b^	–	6.92 ± 0.58	17.66 ± 7.35^###^	< 0.001^b^
HbA1c (%)	7.13 ± 1.18	7.33 ± 1.27	0.564^a^	6.09 ± 1.13	7.10 ± 1.40^***^	8.28 ± 1.45^***^	< 0.001^e^
FPG (mmol/L)	7.33 ± 1.58	8.00 ± 2.35	0.244^a^	5.63 ± 0.83	7.52 ± 2.41^***^	9.21 ± 2.95^***##^	< 0.001^e^
SBP (mmHg)	133.21 ± 9.75	138.04 ± 14.52	0.294^b^	133.90 ± 14.09	131.76 ± 14.16	140.24 ± 15.26^#^	0.019^d^
DBP (mmHg)	75.46 ± 5.98	77.36 ± 6.07	0.960^a^	77.62 ± 8.15	77.66 ± 6.97	75.37 ± 8.53	0.300^d^
HTN, N (%)	13 (54)	12 (43)	0.416^c^	28 (56)	32 (64)	31 (75)	0.150^c^
TC (mmol/L)	5.02 ± 1.41	5.03 ± 1.07	0.989^a^	5.25 ± 1.02	4.91 ± 1.20	4.78 ± 1.03	0.100^d^
TG (mmol/L)	1.14 ± 0.40	1.41 ± 0.59	0.063^a^	1.71 ± 0.88	2.05 ± 1.44	1.81 ± 1.36	0.618^e^
HDL-C (mmol/L)	1.37 ± 0.36	1.41 ± 0.31	0.681^a^	1.42 ± 0.39	1.19 ± 0.30^*^	1.29 ± 0.37	0.011^e^
LDL-C (mmol/L)	3.14 ± 1.24	2.99 ± 0.91	0.625^a^	3.05 ± 0.89	2.78 ± 1.03	2.84 ± 1.00	0.373^d^
ALT (U/L)	19.04 ± 9.57	15.11 ± 5.31	0.232^b^	19.90 ± 12.91	19.82 ± 10.45	19.73 ± 10.82	0.841^e^
AST (U/L)	20.46 ± 5.30	19.21 ± 4.86	0.396^b^	22.42 ± 9.78	20.20 ± 5.64	20.07 ± 6.75	0.467^e^
TBIL (μmol/L)	14.30 ± 8.24	14.51 ± 6.70	0.727^b^	13.54 ± 6.48	14.29 ± 7.58	9.86 ± 3.40^*#^	0.002^e^
BUN (mmol/L)	6.45 ± 2.32	6.25 ± 1.83	0.971^b^	5.50 ± 1.72	5.86 ± 1.41	6.81 ± 2.28^*^	0.009^e^
Scr (μmol/L)	90.29 ± 38.66	79.29 ± 22.33	0.233^b^	79.92 ± 17.31	81.82 ± 21.01	75.00 ± 18.74	0.143^e^
12-HETE (ng/mL)	1.14 ± 0.27	2.12 ± 1.71	0.003^b^	1.25 ± 0.37	1.49 ± 0.70	3.38 ± 2.21^***###^	< 0.001^e^

a, Student’s t-test. b, Mann–Whitney U test. c, Chi-square test. d, ANOVA and LSD post hoc test. e, Kruskal–Wallis H test

^*^P < 0.01, ^**^P < 0.001, ^***^P < 0.0001 when compared with Group Verify-Control

^#^*P* < 0.01, ^##^*P* < 0.001, ^###^*P* < 0.0001 when compared with Group Verify-NDR

BMI, body mass index; HbA1c, glycated hemoglobin; FPG, fasting plasma glucose; SBP, systolic blood pressure; DBP, diastolic blood pressure; HTN, hypertension; TC, total cholesterol; TG, triglyceride; HDL-C, high-density lipoprotein cholesterol; LDL-C, low-density lipoprotein cholesterol; ALT, alanine transaminase; AST, aspartate aminotransferase; TBIL, total bilirubin; BUN, blood urea nitrogen; Scr, serum creatinine

The 12-HETE level in the Test-DR group was significantly higher than that in the Test-NDR group (*P* < 0.01) (Fig. 1B). Binary logistic regression was performed to analyze factors correlated with DR. Independent variables tested in univariate analysis included age, sex (0 = female, 1 = male), BMI, HbA1c, FPG, SBP, DBP, hypertension status (0 = no, 1 = yes), blood lipids, biochemical indices, and 12-HETE. Univariate analysis showed that 12-HETE was associated with the incidence of DR. After adjusting for other significant predictors (triglyceride and alanine aminotransferase,* P* < 0.10) in the multivariate model, only 12-HETE (OR 9.26, 95% CI 1.77–48.59, *P* = 0.008) was significantly associated with DR (Table S2).

In the verification set, the 12-HETE level in the Verify-DR group was significantly higher than that in the Verify-NDR group (*P* < 0.0001) and the Verify-Control group (*P* < 0.0001). There was no significant difference between the latter two (Fig. 1C). According to the results of the univariate analysis, the independent variables entered into the multivariate logistic analysis model were 12-HETE, diabetes duration, HbA1c, FPG, SBP, total bilirubin, and urea. Multivariate analysis confirmed that 12-HETE (OR 10.49, 95% CI 3.23–34.05, *P* < 0.001), diabetes duration (OR 1.33, 95% CI 1.08–1.63, *P* = 0.007) and FPG (OR 1.38, 95% CI 1.01–1.90, *P* = 0.046) were independently associated with DR (Table S3).

## Discussion

Compared with T2DM, the incidence of complications such as DR is lower in patients with childhood-onset T1DM [22]. A 5-year follow-up study found that the prevalence of DR was 19.2% in children with T1DM and 24.6% in those with T2DM [23]. The children in our study were all T1DM patients. We found that significantly higher serum 12-HETE level was related to the incidence of DR in T1DM children, independent of blood glucose, blood pressure, blood lipids, and diabetes duration.

The results of the adult study were consistent with those of the child cohort. Subjects in the Test-NDR group were those who had never developed DR in spite of a diabetes duration of ≥ 20 years. Theoretically, the longer the duration, the more likely DR becomes. In most patients, DR occurs 10–15 years after diabetes diagnosis [24]. It is estimated that the prevalence of DR in patients who have T2DM for ≥ 20 years is 60% [25]. Therefore, there could be some unknown protective factors preventing DR onset in the Test-NDR group. This is why we set up such a group. Patients with newly diagnosed DR in the Test-DR group had a shorter diabetes duration and could represent the general DR population. Logistic regression analysis found that 12-HETE was independently associated with the presence of DR, indicating that lower 12-HETE levels may be a protective factor against DR in diabetic patients. To verify these results, we set up a verification set, whose three groups were more representative of the community population in the real world. The Verify-DR group consisted of T2DM patients with preexisting DR, which could represent the common DR patients. Patients in the Verify-NDR group had a duration < 20 years. They had not yet but may develop DR in future and may not have the protective factors that the Test-NDR group possessed. The 12-HETE level in the Verify-DR group was significantly higher than that in the Verify-NDR group and the Verify-Control group. Subsequent regression analysis suggested that 12-HETE was independently related to DR, as were diabetes duration and FPG, which had been previously known. In the adjusted multivariate model, HbA1c did not show a significant association with DR, possibly due to the small sample, potential confounders or selection bias. In conclusion, the results of the verification set further confirmed that serum 12-HETE has a strong correlation with the prevalence of DR, which has nothing to do with known risk factors.

As the primary enzymes to generate 12-HETE, 12-LOX and 12/15-LOX are widely present in various cells, such as leukocytes and epithelial cells. Both have low levels in peripheral blood [26]. It is undoubtedly difficult to obtain tissue samples to detect LOX content in population studies. Only one study reported that the levels of 12-LOX and 12/15-LOX proteins in the retina of 2 diabetic patients were elevated, but no specific data [12]. The expression of 12/15-LOX is upregulated in human retinal microvascular endothelial cells incubated with high glucose, which further confirms the close relationship between 12/15-LOX and DR [27]. As a small-molecule lipid metabolite, 12-HETE can freely pass through the blood–retina barrier and induce oxidative stress through NADPH oxidase and other pathways, triggering retinal inflammation and angiogenesis [13, 27–29].

This study found no correlation between 12-HETE and any clinical characteristics, such as age, sex, HbA1c, blood pressure, and blood lipids, indicating that serum 12-HETE may not be affected by these factors. AA is the most important ω-6 PUFA in the human retina. Its amount in the body depends on the daily dietary intake [30, 31]. The ratio of ω-3- to ω-6 PUFAs determines the anti-/proinflammatory retinal environment, with increased intake of ω-3 PUFAs reducing the risk of pathological angiogenesis [32]. The PREDIMED trial found that after 6 years, the incidence of vision-threatening DR was reduced by 48% in middle-aged and elderly T2DM patients who adhered to the Mediterranean diet (ω-3 PUFA ≥ 500 mg/d) [33]. Conversely, increased intake of ω-6 PUFAs (e.g., traditional Western diets) leads to the predominance of proinflammatory and proangiogenic effects of their downstream metabolites, promoting the occurrence of cardiovascular diseases, cancer, and autoimmune diseases [31]. Based on this, we infer that the elevated serum 12-HETE level observed in the DR population in this study was probably related to the higher dietary intake of AA.

Our study has some limitations. First, the population was small, mainly because the COVID-19 outbreak hindered follow-up. As a result of it, there was some imbalance in the basic characteristics of the verification set. Second, this study did not conduct research specifically on AA in dietary components. Third, this study only detected the 12-HETE level at the 2021 follow-up, with the level before DR onset unknown, making longitudinal comparisons impossible. We may continue to detect 12-HETE concentrations in future follow-up, for the exploration of the relationship between 12-HETE and the incidence of DR. Fourth, we included a group of healthy controls in the adult study, but not in the children study. It would be meaningful to investigate whether the levels of 12-HETE in health children differ from those with T1DM in future study. Fifth, the subjects were T1DM children and middle-aged and elderly people with T2DM in Shanghai, so the findings cannot be extrapolated to other populations.

The study also has some advantages. This is the first study to report the correlation between serum 12-HETE and the prevalence of DR in children with diabetes. Second, the children in the Children-DR group were from the T1DM population, who are relatively less likely to develop DR, and the adults in the Test-NDR group were from the T2DM population with a long course of disease (≥ 20 years) but who still did not have DR. Setting up such two groups makes more convincing the relationship between 12-HETE and DR. In addition, in the adult study, we adopted an innovative strategy to replicate the results from the test set in the verification set, giving the results more universal significance.

In summary, our study found that serum 12-HETE was positively correlated with the risk of DR in T1DM children and T2DM adults, and it may be an independent risk factor for DR. 12-HETE is also closely associated with other vascular complications of diabetes [34, 35]. Therefore, it will be of great importance for prevention and early detection of DR to pay attention to serum 12-HETE in diabetic population, to explore factors affecting its level, and to clarify the pathogenic processes it participates in.

## Supplementary Information

Below is the link to the electronic supplementary material.

Supplementary file1 (PDF 238 kb)

Supplementary file2 (XLSX 23 kb)

## Data Availability

The datasets generated during and/or analyzed during the current study are available from the corresponding author on reasonable request.
