# Supplementary material for: Increased serum 12-hydroxyeicosatetraenoic acid levels are correlated with an increased risk of diabetic retinopathy in both children and adults with diabetes
Source: Acta Diabetol. 2022 Aug 12;59(11):1505–13. doi: 10.1007/s00592-022-01951-7 (PMC9374295; doi:10.1007/s00592-022-01951-7)
Supplement: Supplementary file 1 — Supplementary file1 (PDF 238 kb) [file 592_2022_1951_MOESM1_ESM.pdf]

Acta Diabetologica

Supplementary Material 1

Increased Serum 12-Hydroxyeicosatetraenoic Acid Levels are Correlated With an Increased Risk of Diabetic Retinopathy in both Children and Adults with Diabetes

Shuli Chen[1], Yu Qian[1], Qiurong Lin[2,3], Zhangling Chen[4,5], Zhaoyu Xiang[1], Lipu Cui[1], Jiaqi Sun[1], Xinran Qin[1], Yi Xu[2], Lina Lu[2] and Haidong Zou[1,2,3,6]

[1] Department of Ophthalmology, Shanghai General Hospital, Shanghai Jiao Tong University, School of Medicine, Shanghai, China

[2] Shanghai Eye Disease Prevention and Treatment Center/Shanghai Eye Hospital, Shanghai, China

[3] Shanghai Engineering Center for Precise Diagnosis and Treatment of Eye Diseases, Shanghai, China

[4] Department of Ophthalmology, Shanghai General Hospital of Nanjing Medical University, Shanghai, China

[5] Department of Ophthalmology, Shanghai Songjiang District Central Hospital, Shanghai, China,

[6] National Clinical Research Center for Eye Diseases, Shanghai, China

Corresponding author:

Haidong Zou

Department of Ophthalmology

Shanghai General Hospital, Shanghai Jiao Tong University, School of Medicine

No.100 Haining Road, Shanghai 200080, China

Tel: +86-21-63240090

Email: zouhaidong@sjtu.edu.cn

Table S1 Logistic regression analyses of potential risk factors for DR in T1DM children

	Univariate logistic regression		
	OR	95% CI	*P*-value
12-HETE (pg/mL)	1.06	1.00–1.13	0.041
Age (years)	0.81	0.56–1.18	0.278
Sex	6.00	0.54–67.28	0.146
BMI (kg/m^2)	0.70	0.42–1.17	0.172
T1DM duration (years)	1.00	0.69–1.44	0.978
HbA1c (%)	0.88	0.38–2.02	0.758
SBP (mmHg)	1.00	0.92–1.08	0.919
DBP (mmHg)	1.08	0.97–1.20	0.160
TC (mmol/L)	1.84	0.53–6.35	0.334
TG (mmol/L)	1.22	0.35–4.31	0.756
HDL-C (mmol/L)	0.96	0.12–7.42	0.968
LDL-C (mmol/L)	2.30	0.50–10.58	0.286

BMI, body mass index; HbA1c, glycated hemoglobin; SBP, systolic blood pressure; DBP, diastolic blood pressure; TC, total cholesterol; TG, triglyceride; HDL-C, high-density lipoprotein cholesterol; LDL-C, low-density lipoprotein cholesterol

Table S2 Logistic regression analyses of potential risk factors for DR in T2DM adults in the test set

	Univariate			Multivariate		
	OR	95% CI	*P*-value	OR	95% CI	*P*-value
12-HETE (ng/mL)	9.26	1.77–48.59	0.008	9.26	1.77–48.59	0.008
Age (years)	1.03	0.96–1.10	0.491			
Sex	0.62	0.21–1.86	0.393			
BMI (kg/m^2)	1.20	0.96–1.49	0.111			
HbA1c (%)	1.15	0.73–1.81	0.556			
FPG (mmol/L)	1.19	0.89–1.58	0.243			
SBP (mmHg)	1.03	0.99–1.08	0.177			
DBP (mmHg)	1.06	0.96–1.16	0.260			
HTN	0.64	0.21–1.90	0.417			
TC (mmol/L)	1.00	0.64–1.57	0.989			
TG (mmol/L)	2.85	0.90–9.00	0.075	–	–	0.067
HDL-C (mmol/L)	1.43	0.27–7.56	0.674			
LDL-C (mmol/L)	0.88	0.53–1.47	0.617			
ALT (U/L)	0.93	0.86–1.01	0.079	–	–	0.120
AST (U/L)	0.95	0.85–1.06	0.377			
TBIL (μmol/L)	1.00	0.93–1.08	0.917			
BUN (mmol/L)	0.95	0.73–1.25	0.723			
Scr (μmol/L)	0.99	0.97–1.01	0.230			

BMI, body mass index; HbA1c, glycated hemoglobin; FPG, fasting plasma glucose; SBP, systolic blood pressure; DBP, diastolic blood pressure; HTN, hypertension; TC, total cholesterol; TG, triglyceride; HDL-C, high-density lipoprotein cholesterol; LDL-C, low-density lipoprotein cholesterol; ALT, alanine transaminase; AST, aspartate aminotransferase; TBIL, total bilirubin; BUN, blood urea nitrogen; Scr, serum creatinine

Table S3 Logistic regression analyses of potential risk factors for DR in T2DM adults in the verification set

	Univariate			Multivariate		
	OR	95% CI	*P*-value	OR	95% CI	*P*-value
12-HETE (ng/mL)	25.61	7.27–90.16	<0.001	10.49	3.23–34.05	<0.001
Age (years)	1.01	0.95–1.07	0.777			
Sex	0.78	0.34–1.82	0.569			
BMI (kg/m^2)	0.90	0.79–1.03	0.127			
T2DM duration (years)	1.40	1.22–1.62	<0.001	1.33	1.08–1.63	0.007
HbA1c (%)	1.83	1.28–2.60	0.001	–	–	0.780
FPG (mmol/L)	1.28	1.07–1.54	0.008	1.38	1.01–1.90	0.046
SBP (mmHg)	1.04	1.01–1.07	0.010	–	–	0.233
DBP (mmHg)	0.96	0.91–1.02	0.163			
HTN	1.74	0.70–4.36	0.235			
TC (mmol/L)	0.90	0.62–1.31	0.594			
TG (mmol/L)	0.88	0.64–1.20	0.423			
HDL-C (mmol/L)	2.30	0.66–8.05	0.194			
LDL-C (mmol/L)	1.09	0.70–1.60	0.788			
ALT (U/L)	1.00	0.96–1.04	0.968			
AST (U/L)	1.00	0.93–1.07	0.921			
TBIL (μmol/L)	0.84	0.75–0.94	0.002	–	–	0.449
BUN (mmol/L)	1.33	1.04–1.70	0.024	–	–	0.469
Scr (μmol/L)	0.98	0.96–1.00	0.115			

BMI, body mass index; HbA1c, glycated hemoglobin; FPG, fasting plasma glucose; SBP, systolic blood pressure; DBP, diastolic blood pressure; HTN, hypertension; TC, total cholesterol; TG, triglyceride; HDL-C, high-density lipoprotein cholesterol; LDL-C, low-density lipoprotein cholesterol; ALT, alanine transaminase; AST, aspartate aminotransferase; TBIL, total bilirubin; BUN, blood urea nitrogen; Scr, serum creatinine
